# Impact of MenAfriVac on Meningococcal A Meningitis in Cameroon: A Retrospective Study Using Case-by-Case-Based Surveillance Data from 2009 to 2015

**DOI:** 10.1155/2021/4314892

**Published:** 2021-09-27

**Authors:** Bouba Gake, Bonaventure Babinne Graobe, Bouba Abdouraman, Crescence Satou Ngah, Ahmadou Aissatou, Nelly-Michèle Gake, Elise Claudine Seukap, Elias Nchiwan Nukenine, Alain G. Etoundi Mballa, Marie-Christine Fonkoua, Jean-Pierre Lombart

**Affiliations:** ^1^Centre Pasteur du Cameroun Annexe de Garoua, Garoua, Cameroon; ^2^Higher Institute of Sciences, Health Technics and Management of Garoua, Garoua, Cameroon; ^3^Faculty of Science, University of Ngaoundere, Ngaoundere, Cameroon; ^4^Ministry of Research and Innovation Technology, Yaoundé, Cameroon; ^5^Department of Disease Control, Epidemics and Pandemics, Ministry of Public Health, Yaounde, Cameroon; ^6^Centre Pasteur du Cameroun, Yaounde, Cameroon

## Abstract

Meningococcal meningitis is a public health concern in Africa. Conjugated vaccine against serogroup A *Neisseria meningitidis* (MenAfriVac) was used in mass vaccination and was proved to have a good impact in the meningitis belt. There is a lack of information about the impact of this intervention in Cameroon after mass vaccination was undertaken. This study aimed at filling the gap in its unknown impact in Cameroon. A retrospective longitudinal study using biological monitoring data of case-by-case-based surveillance for meningitis was obtained from the National Reference Laboratories from 1 January 2009 to 20 September 2015. Immunization coverage data were obtained from Regional Public Health Delegations where immunizations took place. We compared the risks of vaccine serogroup occurrence before and after vaccinations and calculated the global impact using Halloran's formula. Annual cases of meningitis A decreased gradually from 92 in 2011 to 34 in 2012 and then to 1 case in 2013, and since 2014, no cases have been detected. The impact was estimated at 14.48% (*p*=0.41) in 2012 and then at 98.63% (*p* < 0.0001) after the end of vaccinations in 2013. This survey confirms the effectiveness of the MenAfriVac vaccine in Cameroon as expected by the WHO. The surveillance must be pursued and enhanced to monitor coming immunizations measures with multivalent conjugated vaccines for this changing threat.

## 1. Introduction

Cameroon, a central African country with 23 million inhabitants, has experienced epidemic meningococcal meningitis as other countries of the “African meningitis belt,” where *Neisseria meningitidis* (*N. m*) historically caused epidemics [1, 2]. This belt extends from Senegal at the West to Ethiopia at the East, including 26 countries, with about 450 million inhabitants exposed to the risks of epidemics [1, 2]. The main serogroups of *N. meningitis* involved in epidemics in Africa are the following: serogroup A (*N. m* A), W (*N. m* W), X (*N. m* X), and C (*N. m* C) [1, 3–5]. *N. m* A is the one that caused the largest epidemics between 1995 and 2013 [6–13].

Cerebrospinal meningitis epidemics mainly affected four regions of Cameroon: in 1992 in the Far-North region with 8046 cases and 968 deaths. In the North region, epidemics occurred in 1993 with 1190 cases and 136 deaths; and in 1998 there were 2054 cases and 225 deaths [14]. In 2010, the Adamawa region recorded 126 cases. The etiological agent identified in most cases was *N. m* A [7, 10]. With the evolution of the climate change, the Northwest region that was not part of this belt is currently included.

Insufficiently documented epidemics occurred in these regions of Cameroon: Northwest (2001 and 2004), North (2004 and 2011), and Far-North (2007, 2009, and 2011) [14].

Since the cerebrospinal meningitis was a preventable disease, purified polysaccharide based-vaccines available were less effective, could not produce long lasting immunity (young children below 2 years of age were not protected), and were not able to induce “herd immunity” in the population. Despite the use of these vaccines for the control of these epidemics, *N. m* A still caused epidemics [15]. The association of this particular serogroup with the genesis of explosive epidemics has aroused a renewed interest in the search for an effective vaccine against this serogroup. This has led to the Meningitis Vaccine Project (MVP), which was created and permitted to manufacture the MenAfriVac (polysaccharide of group A conjugated to the tetanic toxoid), whose goal was to eliminate meningitis A as a public health problem in Africa [16, 17]. Prequalified by the World Health Organization (WHO) in 2010, this vaccine has been used for the first time in mass vaccination campaigns in 2010 in Burkina Faso, Mali, and Niger.

**Figure 1:**
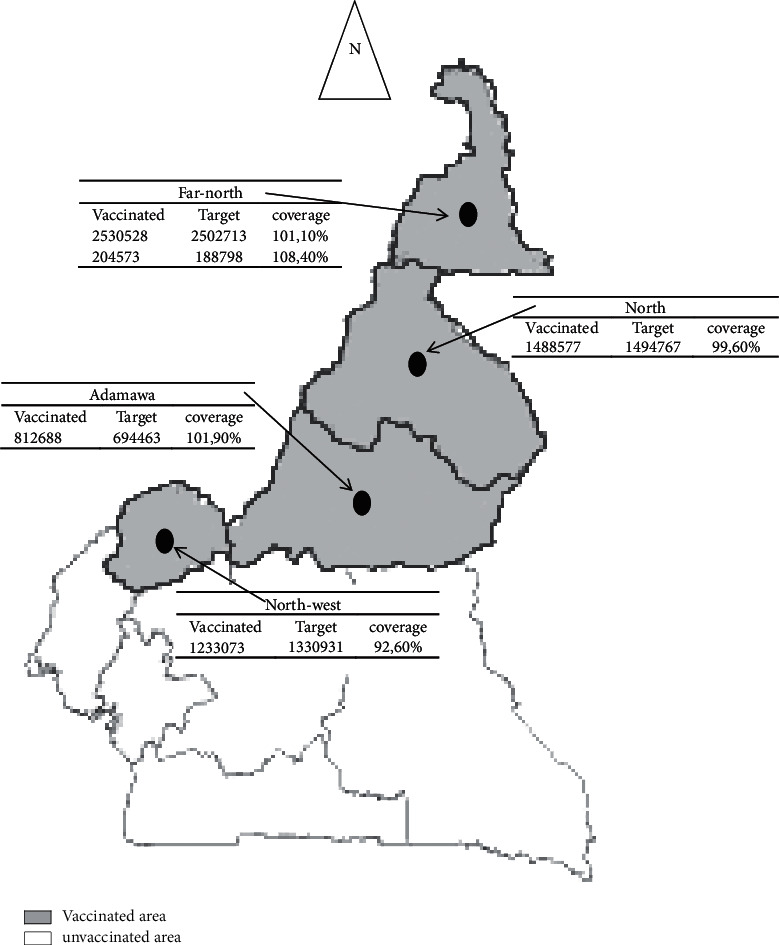
Vaccine coverage in vaccinated regions (North, Far-North, Adamawa, and Northwest) during 2011 and 2012 MenAfriVac vaccination campaigns in Cameroon.

More than 6 million Cameroonians between the ages of 1 and 29 years have received a dose of this vaccine in regions with the largest risk of meningitis epidemics. This vaccination campaign was carried out in two phases: the first phase in the North and Far-North regions from 6 to 12 December 2011 and the second phase in the Adamawa and Northwest regions from 3 to 12 December 2012. Administrative vaccine coverage is reported on the graph in Figure 1.

The impact of this vaccine has been evaluated in some countries where these campaigns have been carried out (Burkina Faso, Niger, and Chad), but, in Cameroon, the impact of this intervention is not yet known. The aim of this work was to assess the impact of the MenAfriVac vaccine in Cameroon after its use in mass vaccination campaigns in the Adamawa, Far-North, North, and Northwest regions.

The general objective of this study was to evaluate the prevalence of meningococcus A since the introduction of the MenAfriVac vaccine and to study the impact of the vaccine. Specifically, it aimed to value the prevalence of the *N. m* A and other etiologies of meningitis before and after the introduction of the MenAfriVac, show the interrelationship between the numbers of vaccinated with MenAfriVac and the annual number of cumulated cases of meningitis, and estimate the impact of the campaign of MenAfriVac vaccination on the *N. m* A meningitis.

## 2. Materials and Methods

This longitudinal survey compared the risk of the meningitis A before and after the campaign of MenAfriVac vaccination, from 1 January 2009 to 20 September 2015, where 3109 suspected samples of cerebrospinal fluids (CSFs) were sent to the laboratories of the Center Pasteur of Cameroon (CPC) of Garoua and Yaounde for analysis in the setting of the case-by-case-based biological surveillance of meningitis.

### 2.1. Study Population

The study population was that of the three northern regions of Cameroon (North, Far-North, and Adamawa) and the Northwest region, which is about 9.5 million (9,605,133) inhabitants, spread over an area of 181,463 km^2^. The target population is that of the patients suspected of having meningitis who have undergone lumbar puncture in the health facilities in the regions concerned and the CSFs were sent to the CPC for analysis.

### 2.2. Setting of Survey

Spinal tap was performed on patients suspected of having meningitis in health settings in concerned regions. CSFs were analyzed in health facilities for preliminary tests and then confirmed at the national reference laboratories (CPC Annex of Garoua for the regions of North, Far-North, and Adamawa and the CPC of Yaounde for the Northwest region).

### 2.3. Methods

CSFs were analyzed by classical bacteriological methods: Gram staining, soluble antigen test by Pastorex Meningitis® method, and culture on blood agar supplemented with PolyViteX incubated in an atmosphere enriched with 5% CO_2_. The polymerase chain reaction (PCR) was used for confirmation of cases.

### 2.4. Variables

The variables of our study were date of sampling, residence, health district, region, vaccination status, and bacteriological diagnosis (by test strip, soluble antigen test, culture, or PCR).

The judging criteria chosen were the number of cumulative cases of *N. m* A confirmed by laboratory per year and the prevalence of *N. m* A among *N. m* isolated per year.

### 2.5. Sources of Data Collection

The data were obtained from the line-listing of meningitis cases of CPC, CSF analysis sheets, the biological meningitis surveillance registers, and the follow-up reports of the MenAfriVac vaccination campaign.

### 2.6. Data Analysis

Proportions of meningitis A per year were compared by chi-square using R version 2.13.0. The threshold of significance was for a *P* value ˂0.05. Graphs were set up by using Excel. The impact of vaccination was calculated according to the formula of [18].

### 2.7. Legal and Ethical Considerations of the Study

The study was carried out within a national program of meningitis surveillance on a case-by-case basis, under the direction of the Ministry of Health, and the CPC laboratories were the national reference laboratories. Ethical approval was not required for routine surveillance of meningitis.

## 3. Results

### 3.1. Description of the Data Obtained

From 1 January 2009 to 20 September 2015, a total of 3109 CSF samples were sent to CPC for biological monitoring of meningitis. 2779 CSFs were analyzed at Garoua's laboratory (89.4%) and 330 at Yaounde's (10.6%). Germs identified after analysis for the period from January 2009 to September 2015 are shown in Table 1.

### 3.2. Evolution of the Prevalence of *N. m* A Compared to Other *N. m* Serogroups

A total of 256 *N. m* cases were isolated in this study. The distribution of the serogroups isolated was as follows: 139 *N. m* A (54%), 116 *N. m* W (45.31%), and 1 *N. m* Y (0.39%).

**Figure 2:**
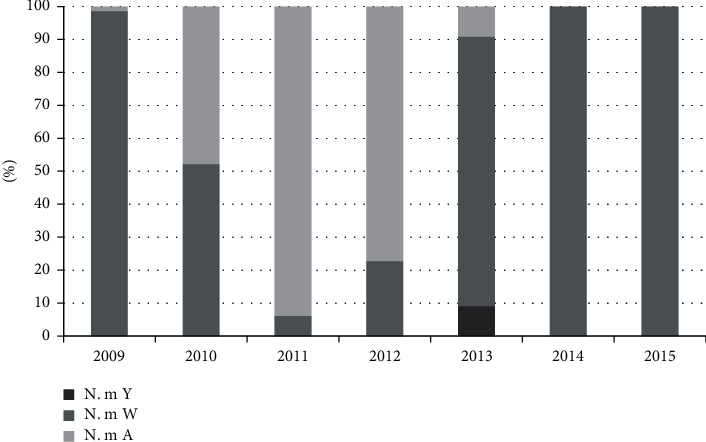
Prevalence of serogroups of *Neisseria meningitidis* in regions vaccinated with MenAfriVac in Cameroon from 2009 to 2015.

The evolution of the prevalence of these serogroups per year is described in the graph in Figure 2. A predominance of serogroup A was observed during the three years before vaccination, followed by a gradual replacement by serogroup W, which became the only one isolated after 2013.

### 3.3. Evolution of the Annual Number of Cumulative Cases of Meningitis A and the Introduction of the MenAfriVac

**Figure 3:**
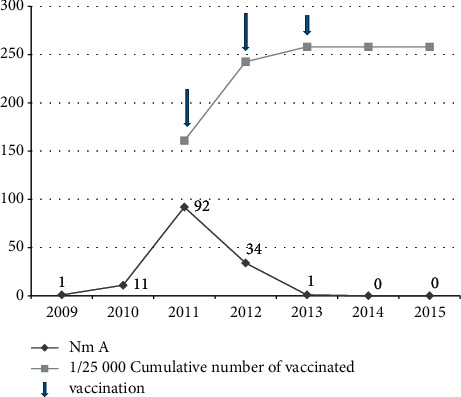
Evolution of the annual number of cumulative cases of meningitis A confirmed and the cumulative number of vaccinated in northern Cameroon from 2009 to 2015.

The annual cumulative cases of meningococcal meningitis A started to increase in mid-2009 and peaked in 2011 and then gradually decreased after the introduction of the MenAfriVac vaccine in December 2011 in the Far-North and North regions and in December 2012 in the Adamawa region. Note that there has not been a case of *N. m* A in the Northwest since the start of surveillance in this area. It can be noticed that, from the year 2014, not a single case of *N. m* A was isolated until the end of the study in September 2015. In addition, the cumulative number of people vaccinated began to increase from the first phase of vaccination in December 2011 and reached its maximum in 2013, the end of the MenAfriVac (last catch-up) vaccination campaign. It can be seen that the curve (Figure 3) of cumulative numbers of vaccinated persons increased progressively after the introduction of the MenAfriVac and then remained constant between 2014 and 2015, while that of the annual number of cumulative cases of *N. m* A decreased progressively during the same period, and then it completely disappeared between 2014 and 2015. Since the introduction of the MenAfriVac, the number of cases of *N. m* A compared to the number of CSFs received during the vaccination period (2011 to 2013) has drastically dropped (*P* < 0.0001) to totally disappear at the end of 2013.

### 3.4. Estimation of Vaccine Global Efficacy from Biological Data according to [18]

The impact of the MenAfriVac vaccination campaign in Cameroon was calculated by considering the CSF biological analysis data from the pre- and postcampaign periods, as described in Table 2. Halloran et al.'s formula was used to calculate this impact [18, 19].

Samples analyzed during the pre-MenAfriVac period (2009–2011) were reference, with an average risk of 0.069. During the per-MenAfriVac period, after the vaccination of December 2011, when 4,019,105 people were vaccinated, this risk was 0.059, a net reduction of 14.48% in the risk of meningitis A (the difference is not significant: *P* value = 0.4118). During the post-MenAfriVac period, after the second phase and the catch-ups that increased the cumulative number of vaccinated population to 6,452,131, the risk was 0.00095, a net reduction of 98.63% (the difference in risk is very significant: *P* value <0.0001).

## 4. Discussion

The vaccination campaign is a public health intervention that must be evaluated to measure its effects and see the achievements made. The aim of this work was to measure the impact of the MenAfriVac vaccination campaign on the occurrence of meningococcal meningitis A in Cameroon. This vaccine has been produced to eliminate this type of meningitis as a public health problem in Africa. Its introduction into Cameroon took place in an epidemiological context characterized by the resurgence of cases of meningitis A in regions at risk. *N. m* A cases were thus consistently isolated during the pre-MenAfriVac period (2009 to 2011). The number of cases drastically decreased after the first phase of introduction of the MenAfriVac in Cameroon and no case was isolated after 2013. The overall impact of this vaccination campaign in Cameroon was 14.48% during the vaccination and 98.63% after vaccination.

This study shows a positive impact of MenAfriVac in Cameroon through the disappearance of meningitis A from vaccinated areas. This effectiveness on the ground confirmed the promises of efficacy that this vaccine foreshadowed. Other authors have reported similar results in other countries: Dougla et al. in 2011 in Chad reported a 90% reduction, and then Gamougam et al. in 2013 reported a reduction of 96%. In the same vein, Collard et al. in Niger in 2012 reported a significant decrease in the prevalence of *N. m* A [20–24].

A prerequisite for the evaluation of any vaccine efficacy is undoubtedly a quality surveillance system capable of detecting suspected cases and ensuring the collection and delivery of the collected CSFs and a microbiological analysis allowing the identification of the etiological agent in accordance with WHO recommendations [25]. Before the introduction of MenAfriVac in Cameroon, epidemiological surveillance of meningitis was part of integrated disease surveillance and response, led by National Health Authority, through the escalation of CSFs from health districts to higher levels and bacteriological analysis at the reference laboratory (CPC) [26, 27]. This monitoring has proved to be unsatisfactory because of insufficient funding for the delivery of samples and analysis. Support from the French Ministry of Foreign Affairs throughout the Special Priority Fund launched in 2007 has revived this system, which has considerably increased the sensitivity of the case detection system, the training of clinicians in lumbar puncture, and the routing of CSFs to the reference laboratory (CPCAG) where molecular techniques (Rt-PCR) for the diagnosis of bacterial meningitis have improved the surveillance system, thus paving the way for the evaluation of the MenAfriVac vaccination campaign announced for 2010 [10]. Case-by-case surveillance, which consisted of detecting and investigating each case, was introduced with the implementation of MenAfriVac in Cameroon in 2011 in order to assess vaccine efficacy [10, 17, 26, 28]. This monitoring was even imperative for a fair measure of vaccine effectiveness. This study is based on the solid scientific basis of this surveillance carried out in the vaccinated regions (North, Far-North, Adamawa, and Northwest). The CSF samples from the first three regions were thus analyzed at the CPCAG and those of the Northwest region at the CPC of Yaounde [10, 21, 26, 28].

Following the first phase of vaccination in 2011 in the North and Far-North regions, administrative vaccination coverage rates were 99.6% and 101.1%, respectively. The establishment of “case-by-case surveillance” to detect suspected cases of meningitis and to investigate confirmed cases of *N. m* A showed that, despite the seemingly satisfactory rate of vaccination coverage (target: ˃95% coverage) in the Far-North and North regions, there have been cases in both regions and there has even been an epidemic in the North. Thus, in the Far-North region, 92 suspect cases and 16 cases of *N. m* A were detected with even cases in vaccinated subjects. In the North region, of 455 suspect cases, 13 cases were *N. m* A with a confirmed epidemic in the Boumba health area in the Poli health district.

Investigations in these districts to evaluate these campaigns revealed shortcomings. The vaccination coverage announced was not real in some areas in both the North and the Far-North regions. In the North, in the health area of Boumba, there was 0% vaccination coverage in Villages Deri and Gagui, and the percentage was 20% in Ngong in the village Laindé Soulédé. This vaccine coverage was below administrative coverage. In Niger, Kim et al. in 2012 showed vaccination coverage lower than administrative coverage, which was even unacceptable (˂70% when the goal was to vaccinate more than 95% of the target population) [29]. Several reasons for nonvaccination in the North region were noted: in particular, the refusal to be vaccinated in the Boumba health district, vaccination being payable in some health districts, and the bad faith of some vaccinators who falsely declared a high number of vaccinates, thus distorting immunization coverage rates. Nonvaccination cases were also reported by Kim et al. in Niger with the following reasons: pregnancy and lactation, absence during the campaign, lack of information, and even refusal to be vaccinated [29].

Despite these constraints, we noted that the annual number of confirmed cases of meningitis A decreased from the pre-MenAfriVac period: from 92 in 2011 to 34 in 2012. There was a significant difference in the number of cases of meningitis A (*P* value 2011/2012 ˂ 0.0001). Corrections made in 2013 in the North and the Far-North regions confirmed the trend observed since the first season. Thus, the number of cases in 2011 decreased to 34 cases in 2012 and then to 1 case in nonvaccinated 11-year-old males, imported in the Far-North in 2013 detected in the refugee camp, and since then not a single case has been registered. This catch-up vaccination had a significant impact on the occurrence of cases (*P* < 0.0001).

For the Adamawa and Northwest, the experience of the two regions previously vaccinated has made it possible to better plan and organize to avoid shortcomings in the North and Far-North regions. This is why as soon as vaccination started in December 2012, no more cases have been detected, whereas epidemics were reported only a few years ago: in 2010 in the Adamawa region [7] and in 2012 in the Northwest region [30]. The Northwest region reported the lowest immunization coverage rates. It was a difficult access area made up of mountains and cliff, which influenced the vaccination and the rate of vaccination coverage.

At the level of Cameroon in general, we found that this vaccination carried out in 2011 and 2012 and the catch-up activities in 2013 have allowed significantly reducing the number of meningitis A cases.

The epidemiology of meningococcal meningitis is dynamic and varies over time [9, 15, 31–35]. To mitigate the effect of these natural changes in the epidemiology of meningococcal meningitis described by Lapeyssonnie in 1963 [1], we compared the risk before and after vaccination intervention and we took as a reference the average risk of occurrence of meningitis A over three years before the start of the vaccination (2009 to 2011). The risk during the postvaccination period (2013 to 2015) was compared with this reference [19, 36]. The risk varied from 6.5% to 0.1%, with a very significant difference (Pearson's Chi-squared test = 68.5012, d*f* = 1, and *P* value <0.0001).

Prior to the advent of MenAfriVac, other polysaccharide vaccines have been widely used to control epidemics of meningococcal meningitis in Africa. The use of these vaccines was less effective because epidemics continued to occur [15]. In Cameroon, vaccination against meningitis was carried out with vaccines of polysaccharide type. Menomune® (a bivalent A + C or tetravalent A + C + Y + W vaccine), used before MenAfriVac, varied with the presence of *N. m* A: lower in well-vaccinated areas and higher in areas badly vaccinated or not vaccinated. There were always cases because this vaccine did not cause a contact effect (protection of people not vaccinated by the vaccinated people who produce IgA in large quantities that they emit with the aerosols that once in the respiratory tracts of the people around them eliminates it). There were cases of meningitis occurring in one household, some of which were vaccinated and others were not. Thus, before the introduction of the MenAfriVac vaccine, there were cases. Since 2009, we noted in the northern part that the number of germs isolated at the national reference laboratory for meningitis oscillated to less than 100 cases per year, with a peak in 2011. The MenAfriVac has the distinction of being very immunogenic and even allows high IgA and IgG levels in the saliva [24, 37–40], which eliminates nasopharyngeal carriage and thus breaks the transmission chain of the germ. This may explain the efficacy found in studies evaluating MenAfriVac.

These vaccination campaigns targeted people aged 1 to 29 years, and this study measured the occurrence of meningitis A among the entire population, including unvaccinated persons. This enabled us to measure the overall effect of the vaccination campaign described by Halloran et al. and Hanquet et al., which included both direct effects (on vaccinated persons) and indirect effects (on unvaccinated persons) [18, 19]. The goal of eliminating meningitis A as a public health problem in Africa is on track to be achieved with MenAfriVac, as did the serogroup C conjugate vaccine of *Neisseria meningitidis* in the United Kingdom [41].

## 5. Conclusion

The aim of this work was to evaluate the effectiveness of the MenAfriVac in Cameroon and to identify the new profile of the etiological agents of meningitis observed after these vaccination campaigns. The results showed that the impact of the MenAfriVac vaccination campaign as organized in Cameroon has progressively increased from 14.48% in 2012 to 98.63% after the campaigns vaccination. The prevalence of *N. m* A varied from 60.97% in 2010 to 93.87% in 2012, and, immediately after the MenAfriVac vaccination campaigns, this prevalence decreased to 11.11% in 2013 and became 0% since 2014, a sign of an overall vaccine effectiveness. However, this disappearance of the strain *N. m* A is giving rise to serogroup W which was already circulating, with *Streptococcus pneumoniae*. *Cryptococcus neoformans* isolated in the CSF analyzed from the immunocompromised subjects remains the seed that must always be sought during a suspicion of meningitis in the immunocompromised subjects.

## Figures and Tables

**Table 1:** Germs isolated from CSF analyzed at Garoua and Yaounde reference laboratories by year and region during surveillance of meningitis in Cameroon from January 2009 to September 2015.

Regions	Germs isolated	Years							
		2009	2010	2011	2012	2013	2014	2015	Total
North	*N. m* A	1	2	57	13	0	0	0	73
	*N. m* W	56	4	6	3	6	2	5	82
	*S. p*	7	8	11	18	8	4	7	63
	*H. i. b*	4	4	1	0	0	0	0	9
	*C. n*	0	0	0	1	2	1	1	5
	Other	1	0	3	0	2	1	1	8

Far-North	*N. m* A	0	1	35	16	1	0	0	53
	*N. m* W	14	8	0	7	0	0	2	31
	*S. p*	4	2	2	9	15	7	0	39
	*H. i. b*	1	0	0	0	0	0	0	1
	Other	0	0	1	0	0	0	0	1

Adamawa	*N. m* A	0	8	0	5	0	0	0	13
	*N. m* W	0	0	0	0	2	0	0	2
	*S. p*	0	1	1	2	3	0	1	8

Northwest	*N. m* A	0	0	0	0	0	0	0	0
	*N. m* W	0	0	0	0	1	0	0	1
	*N. m.* Y	0	0	0	0	1	0	0	1
	*S. p*	0	0	0	0	1	5	0	6
	*C. n*	0	0	0	0	20	10	0	30
	Other	0	0	0	0	0	1	0	1

*N. m*, *Neisseria meningitidis*; *S. p*, *Streptococcus pneunomiae*; *H. i. b*, *Haemophilus influenzae b*; *C. n*, *Cryptococcus neoformans*; A/W/Y, serogroups of *Neisseria meningitidis*.

**Table 2:** Impact of MenAfriVac in Cameroon according to the formula of [18].

	Before vaccination (2009–2011)	During vaccination (2012)	After vaccination (2013–2015)
Total CSF	1491	570	1048
Number of *N. m* A	104	34	1
Risk	0.069751844	0.059649123	0.000954198
Impact (%)	—	14.48	98.63
*P* value	—	0.41	˂0.0001

## Data Availability

The data used to support the findings of the study are available from the corresponding author upon request.
